# Modulation of value-based decision making behavior by subregions of the rat prefrontal cortex

**DOI:** 10.1007/s00213-020-05454-7

**Published:** 2020-02-06

**Authors:** Jeroen P. H. Verharen, Hanneke E. M. den Ouden, Roger A. H. Adan, Louk J. M. J. Vanderschuren

**Affiliations:** 1 Brain Center Rudolf Magnus, Department of Translational Neuroscience, University Medical Center Utrecht, 3584 CG Utrecht, The Netherlands; 2 Department of Animals in Science and Society, Division of Behavioural Neuroscience, Faculty of Veterinary Medicine, Utrecht University, 3584 CM Utrecht, The Netherlands; 3 Helen Wills Neuroscience Institute, Department of Molecular and Cell Biology, University of California Berkeley, Berkeley, CA 94720 USA; 4 Donders Institute for Brain, Cognition and Behaviour, Radboud University, 6525 HR Nijmegen, The Netherlands

**Keywords:** Value, Prefrontal cortex, Decision-making, Reinforcement learning, Behavioral modeling, Rats, Reward, Punishment

## Abstract

**Rationale:**

During value-based decision-making, organisms make choices on the basis of reward expectations, which have been formed during prior action-outcome learning. Although it is known that neuronal manipulations of different subregions of the rat prefrontal cortex (PFC) have qualitatively different effects on behavioral tasks involving value-based decision-making, it is unclear how these regions contribute to the underlying component processes.

**Objectives:**

Assessing how different regions of the rodent PFC contribute to component processes of value-based decision-making behavior, including reward (or positive feedback) learning, punishment (or negative feedback) learning, response persistence, and exploration versus exploitation.

**Methods:**

We performed behavioral modeling of data of rats in a probabilistic reversal learning task after pharmacological inactivation of five PFC subregions, to assess how inactivation of these different regions affected the structure of responding of animals in the task.

**Results:**

Our results show reductions in reward and punishment learning after PFC subregion inactivation. The prelimbic, infralimbic, lateral orbital, and medial orbital PFC particularly contributed to punishment learning, and the prelimbic and lateral orbital PFC to reward learning. In addition, response persistence depended on the infralimbic and medial orbital PFC. As a result, pharmacological inactivation of the infralimbic and lateral orbitofrontal cortex reduced the number of reversals achieved, whereas inactivation of the prelimbic and medial orbitofrontal cortex decreased the number of rewards obtained. Finally, using simulated data, we explain discrepancies with a previous study and demonstrate complex, interacting relationships between conventional measures of probabilistic reversal learning performance, such as win-stay/lose-switch behavior, and component processes of value-based decision-making.

**Conclusions:**

Together, our data suggest that distinct components of value-based learning and decision-making are generated in medial and orbital PFC regions, displaying functional specialization and overlap, with a prominent role of large parts of the PFC in negative feedback processing.

**Electronic supplementary material:**

The online version of this article (10.1007/s00213-020-05454-7) contains supplementary material, which is available to authorized users.

## Introduction

To be able to survive and thrive in a dynamic environment, animals must learn to repeat actions that were profitable in the past, while withholding actions that were not. For example, when a certain action leads to food reward, a hungry animal is likely to repeat that action. Conversely, when an action does not result in expected reward, or when it results in explicit punishment, an animal is likely to avoid that action in the future. This integration of action-outcome relationships is the basis of reinforcement learning theory (Rescorla and Wagner 1972; Sutton and Barto 1998; Dayan and Daw 2008), which states that value is attributed to preceding actions, updated based on their outcomes, and stored for when confronted with a comparable choice later on. Such learning processes enable animals to flexibly adapt to a changing world and use environmental resources optimally (Verharen et al. 2019b).

It has long been known that function of the prefrontal cortex (PFC) underlies such value-based learning and decision-making processes (Miller and Cohen 2001; Dalley et al. 2004; Roberts 2006; Robbins and Arnsten 2009; Floresco 2013). For example, lesions or temporary inactivations of the rodent PFC impair processes like reversal learning (Chudasama and Robbins 2003; Dalton et al. 2016; Izquierdo et al. 2017; Hervig et al. 2019), set shifting (Birrell and Brown 2000), and probabilistic discounting (St Onge and Floresco 2010). Importantly, different subregions of the PFC have been implicated in distinct aspects of value-based learning and decision-making. For example, in humans, functional activity in orbitofrontal regions is crucial for flexible decision-making behavior, while activity in the dorsolateral PFC is important for reward-related feedback sensitivity (Hornak et al. 2004). Likewise, pharmacological inactivation of distinct PFC regions in the rat has been shown to alter performance in a probabilistic reversal learning task in qualitatively different ways (Dalton et al. 2016).

Although conventional measures of performance in operant tasks, including reversal learning, provide important insights into how task behavior is altered by neuronal manipulations, value-based choices are the result of a dynamic process in which outcome expectancies, innate preferences, and explorative urges are weighed, ultimately leading to a choice between different options. As such, alterations in overt behavior are typically the result of changes in a variety of such component processes. One way to gain insights into how neuronal manipulations alter these processes is by performing computational trial-by-trial analysis of the behavioral data. For example, one could assume that animals make decisions by tracking the reward value of different choice options and that they update these values based on the outcome after every trial. By using such models, one can describe behavior on the basis of computations that may be akin to processes that occur within the neural circuit, including reward prediction error-guided learning (Schultz et al. 1997) and balancing exploration versus exploitation (Cohen et al. 2007).

Here, we investigated the anatomical organization of core processes underlying value-based learning and decision-making within the rat PFC. We employed an experimental design that was comparable to a previous study (Dalton et al. 2016) that assessed the effects of pharmacological inactivation of five different rat PFC regions on probabilistic reversal learning, all of which have been implicated in different aspects of value-based behavior (Birrell and Brown 2000; Miller and Cohen 2001; Dalley et al. 2004; Roberts 2006; Robbins and Arnsten 2009; St Onge and Floresco 2010; Floresco 2013; Izquierdo et al. 2017): the anterior cingulate cortex (ACC), the prelimbic cortex (PrL), the infralimbic cortex (IL), the medial orbitofrontal cortex (mOFC), and the lateral orbitofrontal cortex (lOFC). We modified this behavioral paradigm for computational trial-by-trial analysis and used this tool to investigate how inactivations of these same five regions affected the structure of responding in the task. Finally, we show how alterations in these processes may ultimately drive changes in reversal learning performance.

## Materials and methods

### Animals

Sixty adult male (> 300 g) Long-Evans rats (Janvier labs, France) were used for the experiments. Rats were singly housed in a humidity- and temperature-controlled room and were kept on a 12-h/12-h reversed day/night cycle (lights off at 8 AM). All experiments took place in the dark phase of the cycle. Animals were kept on food restriction (~ 5-g standard lab chow per 100-g body weight per day) during behavioral training and testing. All experiments were conducted in accordance with European (2010/63/EU) and Dutch (Wet op de Dierproeven, revised 2014) legislation and were approved by the Dutch Central Animal Testing Committee and by the Animal Ethics Committee and the Animal Welfare Body of Utrecht University.

### Surgeries

Animals were implanted with bilateral guide cannulas above each of the target areas (one brain area per group). For surgery, animals were anesthetized with an i.m. injection of a mixture of 10 mg/kg fluanisone and 0.315 mg/kg fentanyl (Hypnorm, Janssen Pharmaceutica, Beerse, Belgium). Animals were placed into a stereotaxic apparatus (David Kopf Instruments, Tujunga, USA), and an incision was made along the midline of the skull. Using a dental drill, two small craniotomies were made above the area of interest, and 26-G guide cannulas (Plastic Ones, Roanoke, USA) were lowered to the following positions (relative to Bregma; based on Paxinos and Watson’s brain atlas, 6th Edition):

- ACC: AP + 2.0 mm ML ± 0.6 mm DV − 2.2 mm from the skull
- PrL: AP + 3.2 mm ML ± 0.6 mm DV − 2.6 mm from the skull
- IL: AP + 3.2 mm ML ±0.6 mm DV − 4.3 mm from the skull
- mOFC: AP + 4.4 mm ML ± 0.6 mm DV− 3.8 mm from the skull
- lOFC: AP + 3.6 mm ML ± 2.6 mm DV − 3.7 mm from the skull under a 5° angle.

For the ACC, PrL, IL, and mOFC groups, guide cannulas were used with a bilateral protrusion of 5 mm (with 1.2-mm space between the protrusions). For the lOFC group, single cannulas were used with a protrusion length of 5 mm.

Guide cannulas were secured with screws, dental glue (C&B Metabond, Parkell Prod Inc., Edgewood, USA), and dental cement, and the skin of the animals was sutured such that no skull was exposed. After the surgery, animals received saline (10 ml once, s.c.) and carprofen for pain relief (5 mg/kg, 3× daily, s.c.). Dummy injectors were placed into the cannulas. Animals were allowed to recover for at least 7 days before behavioral training started.

### Behavioral task

**Fig. 1:**
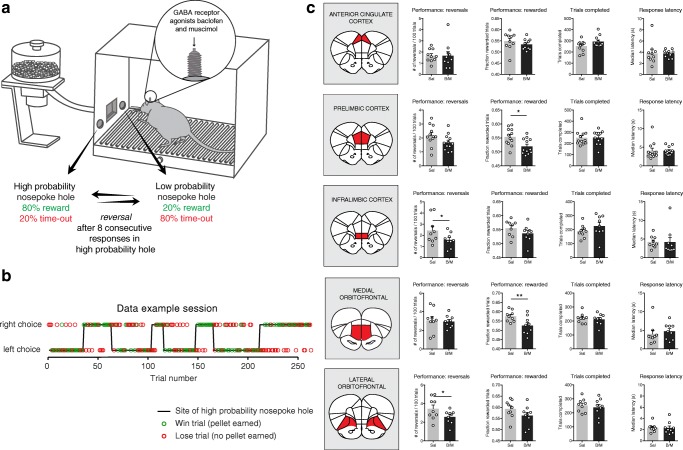
Effects of PFC inactivation on probabilistic reversal learning. **a** Probabilistic reversal learning setup. **b** Example session of one rat. **c** Effects of PFC inactivation on probabilistic reversal learning. ACC, *n* = 10 rats; PrL, *n* = 12 rats; IL, *n* = 9; mOFC, *n* = 9; lOFC, *n* = 9. *P < 0.05, **P < 0.01, ***P < 0.001, ****P < 0.0001 (post hoc Holm-Sidak test; see also the Supplementary statistics table in Online Resource 1; for infusion sites see Online Resource 3)

The behavioral task was conducted in operant conditioning chambers (Med Associates Inc., USA; 30.5 × 24.2 × 21.0 cm), placed within sound-attenuated cubicles. The boxes contained two illuminated nose poke holes, a tone generator, and a house light on one side of the chamber, and on the other side of the chamber a food receptacle delivering 45-mg sucrose pellets (SP; 5UTL, TestDiet, USA) flanked by two cue lights (note that for visualization purposes, the food receptacle and nose poke holes were shown on the same side of the chamber in Fig. 1a; in reality, these were on opposite sides).

At task initiation, one of the two nose poke holes was randomly assigned as the high-probability hole that gave 80% chance on reward and 20% chance on a time-out, and the other hole was assigned as the low-probability hole, which gave 20% chance on reward and 80% chance on a time-out (Fig. 1a, b). Determination of the response outcome (reward or time-out) happened through independent sampling, so that the outcome of the previous trial did not affect the odds of reward in the next trial. The start of the session was signaled to the animal by illumination of the house light and the two nose poke holes.

Directly after a “win” response (i.e., a responses that resulted in reward delivery), the lights in the two nose poke holes were turned off, a sucrose pellet was delivered into the food receptacle, a tone was played for 0.5 s, and the two cue lights next to the food receptacle were turned on. Consumption of the reward was measured by an infrared light sensor in the food receptacle, after which the cue lights were extinguished and a new trial was initiated. After a “lose” response (i.e., a response that resulted in a time-out), the house light and lights in the nose poke holes were turned off, and a 10-s time-out started during which animals remained in the dark, and poking either of the two nose poke holes was without scheduled consequences. After 10 s, a new trial was automatically initiated, signaled to the animal by the illumination of the house light and the two nose poke holes.

When the animals made 8 consecutive responses in the high-probability nose poke hole, the contingencies reversed, so that the previously high-probability nose poke hole became the low-probability nose poke hole, and vice versa. The task automatically terminated after 90 min, and the animals were allowed to make an unlimited number of trials during this period.

This task is a probabilistic reversal learning paradigm (Bari et al. 2010; Dalton et al. 2016) that was changed to be more suitable for behavioral modeling in two different ways. First, the animals were allowed to make an unlimited amount of trials during the 90-min session, as there is a strong positive relation between reliability of model parameter estimation and the amount of trials on which that estimation is based. Second, there was no restriction to the time in which the animals could make a response at one of the nose poke holes (i.e., the task was self-paced, and no trials were designated as “omissions”), because it is unknown how an animal may update the values of the two nose poke holes after an omission (although temporal value decay functions may be used to include this). Importantly, the lack of omissions may mask any attentional deficits evoked by pharmacological inactivation, although this is also partially captured by the “response latency” parameter.

For each trial, the choice of the animal, the side of the high-probability nose poke hole, the outcome of the trial (win or lose), and the timestamps of trial start and nose poke response were monitored. Win-stay was defined as the fraction of win trials on which the animal chose that same nose poke hole on the next trial. Lose-switch was defined as the fraction of lose trials on which the animal chose a different nose poke hole on the next trial.

### Pharmacological inactivations

Infusions took place after the animals reached stable performance in the task, which was defined as a non-significant result of a repeated measures one-way ANOVA on the total number of reversals per 100 trials for 3 consecutive days, which was typically after ~ 10 training sessions. One day before test sessions, all animals received an infusion of saline, to habituate them to the infusion procedure. The next days, animals received an infusion with saline or a mixture of baclofen (1 nmol; Sigma-Aldrich, The Netherlands) and muscimol (0.1 nmol; Sigma-Aldrich, The Netherlands) dissolved in saline, counterbalanced between days, with 24 h in between test sessions.

For the infusions, dummy injectors were removed and replaced by injectors that injected 0.3 μl/side of dissolved drug solution (or saline) at a rate of 0.3 μl/min with a syringe pump (Harvard apparatus, Holliston, USA). The injectors were kept in place for an additional 30 s after the infusion to allow for diffusion of the drug into the tissue. Injectors of the double cannulas protruded 1 mm, and the injectors of the single cannulas protruded 0.4 mm below the termination point of the guide cannula. Subsequent to the infusion, the animals were placed back into their home cage for 10 min, after which they were placed in the operant boxes.

To reduce intra-animal variability, thereby reducing the number of animals necessary to achieve the same statistical power, we performed the experiment a second time and averaged all task measures across the two conditions. In other words, animals were measured twice after saline infusion, and twice after baclofen + muscimol infusion, and the outcomes were averaged to get one single saline and one single baclofen + muscimol measure, which were used for statistical analyses (see also Online Resource 1 for effect sizes of individual measurements).

### Computational modeling

#### Basic model

We fit a series of Q-learning models to our data to assess which model (i.e., task performance strategy) best described the animals’ behavior in the task (Rescorla and Wagner 1972; Verharen et al. 2019a; Verharen et al. 2019c). The first model that we tested is the classic Rescorla-Wagner Q-learning model (RW1) that assumes that on every trial *t*, the nose poke values are updated based on the reward prediction error (RPE), which is the difference between the reward received (this is 1 for win trials, 0 for lose trials) and the reward expected (i.e., the expected value *Q* of the chosen nose poke hole *s*):

1 $$ {\mathrm{RPE}}_t={\mathrm{outcome}}_t-{Q}_{s,t-1} $$

2 $$ \mathrm{so}\ \mathrm{that}\ {\mathrm{RPE}}_t=\Big\{{\displaystyle \begin{array}{cc}1-{Q}_{s,t-1}& \mathrm{for}\ \mathrm{win}\ \mathrm{trials}\\ {}0-{Q}_{s,t-1}& \mathrm{for}\ \mathrm{lose}\ \mathrm{trials}\end{array}} $$

Nose poke hole values were subsequently updated with learning rate α according to a Q-learning rule:

3 $$ {Q}_{s,t}={Q}_{s,t-1}+\alpha \cdot {\mathrm{RPE}}_t $$

Note that the value of the unchosen side was not updated and thus retained its previous value. For the first trial, both nose poke values were initiated at 0.5.

The relationship between nose poke values *Q*_left_ and *Q*_right_, and the probability that the rat chooses the left or right (*p*_left*,t*_, respectively *p*_right*,t*_) nose poke hole in every trial, was described by a Softmax function:

4 $$ {p}_{\mathrm{right},t}=\frac{\exp \left(\beta \cdotp {Q}_{\mathrm{right},t}\right)}{\exp \left(\beta \cdotp {Q}_{\mathrm{left},t}\right)+\exp \left(\beta \cdotp {Q}_{\mathrm{right},t}\right)} $$

and

5 $$ {p}_{\mathrm{left},t}=1-{p}_{\mathrm{right},t} $$

In this function, *β* is the Softmax inverse temperature, which indicates how value-driven the agent’s choices are. If *β* becomes very large, then the value function *β∙Q*_*s,t*_ of the highest valued side becomes dominant, and the probability that the animal chooses that side approaches 1. If *β* is zero, then *p*_left*,t*_ = *p*_right,*t*_ = e^0^/(e^0^ + e^0^) = 0.5. *β* is sometimes referred to as the explore/exploit parameter, where a low *β* favors exploration (i.e., sampling of all options) and a high *β* favors exploitation (i.e., choosing the most beneficial option). Therefore, a sharp decrease in *β* may reflect a more general disruption of behavior, since it indicates that the animal chooses more randomly.

All the subsequently tested models are extensions of this Rescorla-Wagner Q-learning model.

#### Model extensions

The second model we tested (RW2) is similar to RW1, except that separate learning rates were used for learning from positive (reward delivery; win trials) and negative (reward omission; lose trials) feedback, α^+^ and α^−^, respectively. The value updating function is thus given by Eq. 6:

6 $$ {Q}_{s,t}=\Big\{{\displaystyle \begin{array}{cc}{Q}_{s,t-1}+{\alpha}^{+}\cdotp {\mathrm{RPE}}_t& \mathrm{for}\ \mathrm{win}\ \mathrm{trials}\\ {}{Q}_{s,t-1}+{\alpha}^{-}\cdotp {\mathrm{RPE}}_t& \mathrm{for}\ \mathrm{lose}\ \mathrm{trials}\end{array}} $$

Model RW3 is an extension of model RW2 and adds a stickiness parameter *π* to the model. This parameter indicates a preference for the previously chosen (*π* > 0; perseveration) or previously unchosen (*π* < 0; alternation) option, so that the Softmax is given by Eq. 7:

7 $$ {p}_{\mathrm{right},t}=\frac{\exp \left(\beta \cdotp {Q}_{\mathrm{right},t}+\pi \cdotp {\phi}_{\mathrm{right},t}\right)}{\exp \left(\beta \cdotp {Q}_{\mathrm{left},t}+\pi \cdotp {\phi}_{\mathrm{left},t}\right)+\exp \left(\beta \cdotp {Q}_{\mathrm{right},t}+\pi \cdotp {\phi}_{\mathrm{right},t}\right)} $$

Here, *ɸ* is a boolean with *ɸ* = 1 if that hole was chosen in the previous trial, and *ɸ* = 0 if not. For example, if the right nose poke hole was chosen in trial *t-1*, then *ɸ*_right,*t*_ becomes 1, and *ɸ*_left,*t*_ becomes 0. This adds a certain amount of *π* to the value function of the right nose poke hole in trial *t*, in addition to the nose poke hole’s expected value *Q*_right,*t*_.

In addition, we tested a hybrid Rescorla-Wagner/Pearce-Hall model of reinforcement learning that is able to account for an increased learning rate when task volatility is higher, for example right after a reversal. As such, it has a fixed single learning rate *α*, and a variable learning rate *γ* that is dependent on the unsigned prediction error to a certain amount *η* (which was a free variable in the model).

Online Resource 2 shows the equations that are used for value updating and the conversion of values into action probabilities for each of the models.

### Parameter estimation

Modeling was performed using Matlab (Version R2018a; MathWorks Inc., USA). To obtain realistic estimates of the model parameters on a population level, we used maximum a posteriori probability (MAP) estimation. This was done because a simple grid search sometimes lead to unrealistic parameter values (for example, learning rates > 1). The used priors for the MAP estimation were:

- α^+^, α^−^: betapdf(1.5, 1.5)
- π: normpdf(0.5, 0.5)
- β: normpdf(2, 2)

Multiplication of these priors with the likelihood gives the posterior probability of the model parameters given the observed choice sequence:

8 $$ P\left(\left\{{\alpha}^{+},{\alpha}^{-},\pi, \beta \right\}|\mathrm{data},\mathrm{model}\right)=P\left(\mathrm{data}|\mathrm{model},\left\{{\alpha}^{+},{\alpha}^{-},\pi, \beta \right\}\right)\cdotp P\left(\left\{{\alpha}^{+},{\alpha}^{-},\pi, \beta \right\}|\mathrm{model}\right) $$

in which *P*(data | model, {α^+^, α^−^, π, β}) is the likelihood of the observed choice sequence (from trial 1 to the last trial *T*) given the model and the parameter settings (computed as the log likelihood):

9 $$ \log \left(P\left(\mathrm{data}|\mathrm{model},\left\{{\alpha}^{+},,,{\alpha}^{-},,,\pi,,, \beta \right\}\right)\right)=\overset{T}{\sum \limits_{t=1}}\log \left(P\left({\mathrm{choice}}_t|{Q}_{left,t},{Q}_{right,t},{\phi}_{left,t},{\phi}_{right,t}\right)\right) $$

The posterior probability was calculated for many combinations of parameters {α^+^, α^−^, π, β} and arranged in a multidimensional grid. Best-fit parameter values were then estimated by integrating these posterior probabilities over the parameter’s range, marginalized over the other parameters.

### Model comparisons

The log-model evidences of individual sessions were penalized for model complexity by computing the Akaike information criterion and Bayesian information criterion:

10 $$ \mathrm{AIC}=2\times \left[\mathrm{number}\ \mathrm{of}\ \mathrm{free}\ \mathrm{parameters}\ \mathrm{in}\ \mathrm{the}\ \mathrm{model}\right]-2\times \log\ \left(\mathrm{likelihood}\right) $$

11 $$ \mathrm{BIC}=-2\times \log\ \left(\mathrm{likelihood}\right)+\left[\mathrm{number}\ \mathrm{of}\ \mathrm{free}\ \mathrm{parameters}\ \mathrm{in}\ \mathrm{the}\ \mathrm{model}\right]\times \log\ \left(\left[\mathrm{number}\ \mathrm{of}\ \mathrm{trials}\right]\right) $$

As such, a lower value of the AIC and BIC reflects more evidence in favor of the model. In addition, model comparisons contained a random choice model, in which all choices had a probability of 0.5; hence, the log likelihood for each session was computed as log(0.5^total trials^). To compare models, we entered the AICs of all baseline sessions (i.e., after saline infusion) in a random effects Bayesian model comparison (implemented in SPM12) analysis to assess the evidence that one model is more likely than any of the others (see Rigoux et al. 2014).

### Statistical analysis

Statistical tests were performed with Prism 6 (GraphPad Software Inc.). For each measure, a 2-way repeated measures analysis of variance (ANOVA) was used, in which drug (saline versus baclofen + muscimol) was used as a within-subject repeated measures factor, and group (ACC, PrL, IL, mOFC, or lOFC) as a between-subjects factor. When the ANOVA yielded a significant interaction effect, or a main effect of drug (*p* < 0.05), a planned pairwise comparison (Holm-Sidak multiple comparisons test) was used to test, for each group, whether there was a significant difference between the saline and baclofen + muscimol sessions, similar to Hervig et al. (2019). All statistics are presented in the Statistics Table in Online Resource 1. In all figures, *P < 0.05, **P < 0.01, ***P < 0.001, and ****P < 0.0001.

### Histology

After the experiments, brains were sliced using a cryostat (50-μm slices), and histological verification of infusion sites was conducted by an experimenter blind to the outcome of the experiments (for histological placements, see Online Resource 3). All experimental groups started with 12 animals. The following animals were excluded from the analysis:

- ACC group: 1 rat died during surgery, and 1 rat was excluded due to misplacement of the cannulas (final group, *n* = 10 rats).
- PrL group: none (final group, *n* = 12 rats).
- IL group: 2 rats died during surgery, and 1 rat was excluded due to misplacement of the cannulas (final group, *n* = 9 rats).
- mOFC group: 2 rats died during surgery, and 1 rat was excluded due to misplacement of the cannulas (final group, *n* = 9 rats).
- lOFC group: 1 rat died during surgery, and 2 rats were excluded due to misplacement of the cannulas (final group, *n* = 9 rats).

## Results

### Behavioral probabilistic reversal learning measures

A two-way repeated measures ANOVA, using brain region as a between-subject factor and inactivation as a within-subject factor, showed a main effect of inactivation on both the number of reversals and the fraction of rewarded trials, but no significant brain region × inactivation interaction effect for both these measures, suggesting that inactivation of the regions has comparable effects on performance (Fig. 1c; see also Supplementary Statistics table in Online Resource 1). Yet, planned pairwise comparisons, testing the difference between inactivation (B/M) condition and control (saline) condition using the Holm-Sidak test, showed only a significant reduction in the fraction of rewarded trials after PrL and mOFC inactivation. In contrast, IL and lOFC inactivation did not change the number of rewards earned, but did result in a significant reduction in the total number of reversals the animals achieved (i.e., the rats less often reached the criterion of 8 consecutive responses at the high-probability nose poke hole). Despite the fact that this did not lead to explicit negative consequences for the animals (i.e., less rewards earned), a reduction in the total number of reversals indicates lower task volatility (as reward contingencies switched less often), which might be easier for the animals and this may therefore mask a reduction in performance. In addition, inactivation of the ACC did not significantly affect any of the two performance measures. Finally, further analyses of the data demonstrated that inactivation of none of the regions changed the number of trials completed in the task or response latencies (Fig. 1c).

### Computational model parameters

**Fig. 2:**
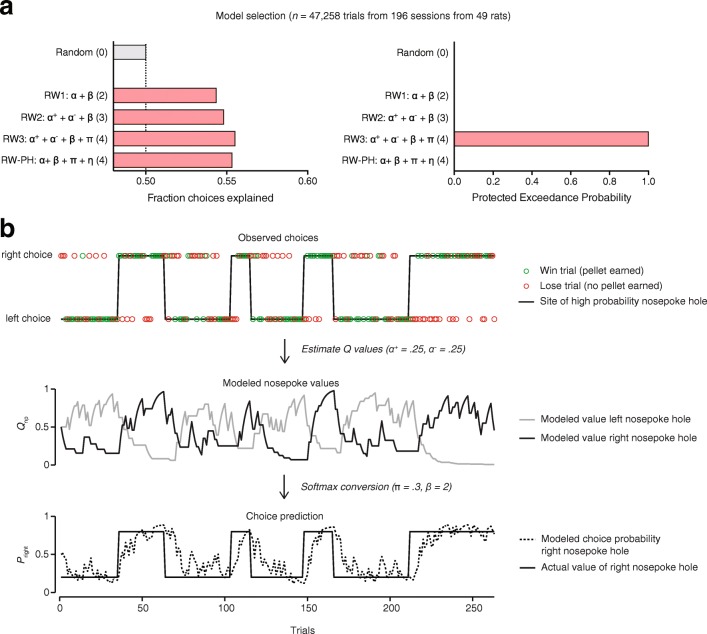
Behavioral model selection. **a** We fit several reinforcement learning models to our data and estimated which model (i.e., strategy) best described the animals’ behavior. Numbers in parentheses refer to the number of free parameters in the model (see also Online Resources 2 (model equations), 4 (table of model selection), and 5 (model selection per inactivation condition)). **b** The “winning” model was a Rescorla-Wagner model (RW3), in which the animals track the value of both nose pokes over an extended history of outcomes by learning from reward and punishment (i.e., reward versus reward omission)

To gain insight into the component processes subserving reversal learning that are disrupted by the pharmacological inactivations, we fit a series of Q-learning models to the data. These models assume that the animals perform the task in order to maximize reward, by using past outcomes to track the value of each of the two nose poke holes and make choices based on these stored values. To estimate which of the learning mechanisms best described the animals’ behavior, we fit different reinforcement learning models to all 196 individual reversal learning sessions and performed random effects model selection using the log model evidence estimates (Rigoux et al. 2014) (Fig. 2a). The first model we tested is the classic Rescorla-Wagner Q-learning model, in which the value of each choice option is updated according to the prediction error (Rescorla and Wagner 1972), i.e., the difference between the expected outcome and the actually received outcome according to learning rate *α*. Considering that a wealth of literature implicates the PFC in value-based learning and decision-making (Miller and Cohen 2001; Dalley et al. 2004; Roberts 2006; Robbins and Arnsten 2009; Floresco 2013), this model should be able to explain the impairments in reversal learning caused by the PFC inactivations. We next extended this model in various ways. Model 2 included separate learning rates for negative and positive feedback, *α*^*+*^ and *α*^*−*^, since certain manipulations only impact one type of feedback learning (Verharen et al. 2018). In model 3, we added a stickiness parameter *π* to this second model to assess the degree to which an animal perseverates on one choice option, independent of prior outcomes (Gershman 2016). Model 4 was a Rescorla-Wagner/Pearce-Hall hybrid model (Pearce and Hall 1980; Li et al. 2011) which was used to assess whether the learning rate changes when task volatility is higher (i.e., in proportion to the absolute prediction error, for example after a reversal). For all models, the value estimates were converted to choice probabilities using a Softmax function, allowing choice behavior to be stochastic to an extent described by parameter 1/*β* (often called the explore/exploit parameter; see “Materials and methods” section).

**Fig. 3:**
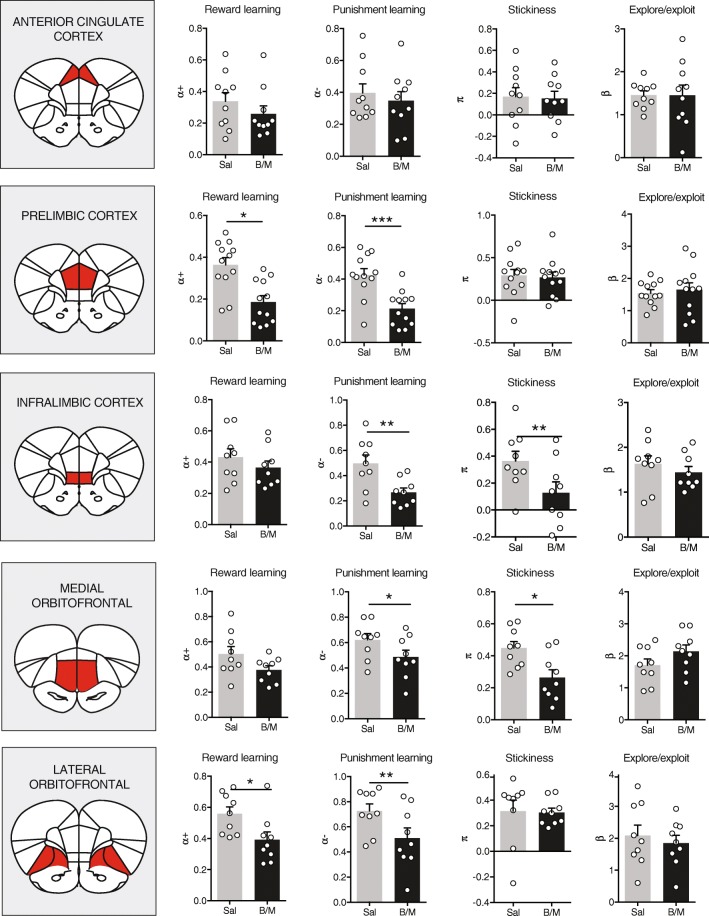
Model coefficients. Best-fit model parameters for each session. Inactivation of the PrL and lOFC impaired reward and punishment learning, whereas inactivation of the IL and mOFC impaired punishment learning and reduced choice perseveration (i.e., repeated choices for the same nose poke hole). ACC, *n* = 10 rats; PrL, *n* = 12 rats; IL, *n* = 9; mOFC, *n* = 9; lOFC, *n* = 9. *P < 0.05, **P < 0.01, ***P < 0.001 (post hoc Holm-Sidak test; see also Online Resource 1)

Model 3 provided the best fit to the data (protected exceedance probability = 1; see Fig. 2a and Online Resource 4); it explains the behavior of the animal on the basis of reward (i.e., positive feedback: reward delivery) and punishment (i.e., negative feedback: time-out instead of reward) learning rates *α*^+^ and *α*^−^, stickiness parameter *π*, and stochasticity parameter *β* (Fig. 2b). Assessing the parameter values as a function of inactivation condition revealed differential contributions of the PFC subregions to these different computational building blocks of value-based decision-making (Fig. 3). A two-way ANOVA revealed a main effect of inactivation condition on positive and negative feedback learning, but no inactivation × brain region interaction effect (see Supplementary Statistics table in Online Resource 1), suggesting a general impairment in integrating past outcomes after inactivation of one of the PFC regions. Yet, post hoc planned comparisons using the Holm-Sidak test showed only a significant reduction in positive feedback learning after inactivation of the PrL and lOFC, and a reduction in negative feedback learning after inactivation of the PrL, IL, mOFC, and lOFC, but not ACC. In contrast to these two learning parameters, a significant inactivation × brain region interaction effect was observed for the stickiness parameter, suggesting that stickiness is differentially affected by inactivation of the different brain regions. Planned comparison Holm-Sidak tests showed that a significant reduction in stickiness was observed after inactivation of the IL and mOFC, with no effects after ACC, PrL, and lOFC inactivation. Importantly, estimates of stochasticity parameter *β* were unchanged across the inactivations, suggesting that pharmacological inactivation of the PFC affected value-based *learning* rather than value-based *decision-making*.

**Fig. 4:**
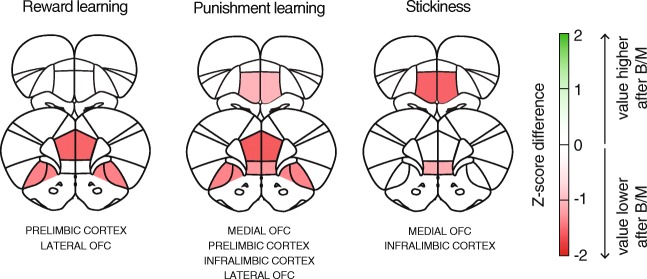
Visual summary. PFC subregions have distinct, albeit overlapping, functions in value-based behaviors. All regions except the ACC are involved in punishment learning. Shown is *Z*-score of B/M effect ((mean_BM_ − mean_Sal_)/SD_sal_)

Interestingly, when we perform Bayesian model selection for each inactivation condition separately, it can be seen that in most cases, model 3 remains the best-fit model after pharmacological inactivation (Online Resource 5), indicating that the inactivation-induced changes are of quantitative, rather than qualitative in nature. An exception may be inactivation of the mOFC, after which the Rescorla-Wagner-Pearce-Hall model (model 4) is slightly favored over the others. See Fig. 4 for a visual summary of the inactivation-induced changes in computational model parameters, as indicated by post hoc Holm-Sidak tests.

### Data simulations

**Fig. 5:**
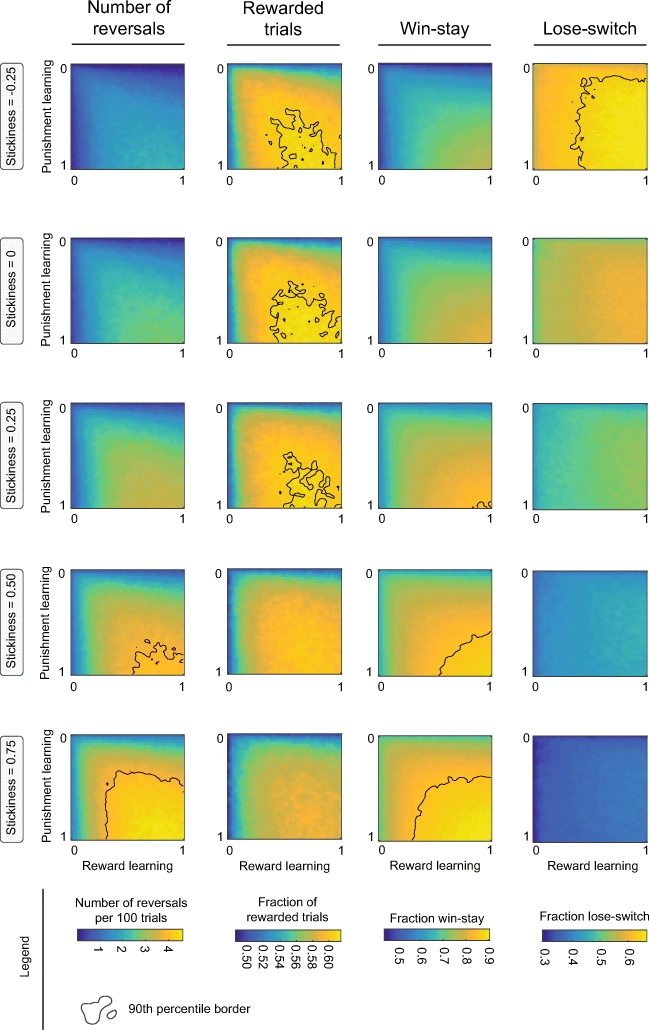
Simulated data. We simulated probabilistic reversal learning sessions (50 simulations per condition (i.e., per pixel), 200 trials per session), to assess how changes in the computational model parameters affect conventional measures of task performance in the (simulated) data. Explore/exploit parameter *β* was fixed at the animals’ grand average from the experimental data (*β* = 1.686). The highest number of reversals can be obtained by a combination of high learning and high stickiness, whereas the number of rewarded trials could be maximized by having high learning and an intermediate value of the stickiness parameter. Win-stay/lose-switch measures were mostly dependent on the stickiness parameter, but can be modulated by both reward and punishment learning. Thus, whether changes in the computational model parameters lead to significant changes in conventional task measures is highly dependent on the baseline behavior of the animal and size and direction of the effects.

Although the effects of inactivation of different PFC regions had similar effects on the computational model parameters (e.g., IL and mOFC inactivation both decrease punishment learning and stickiness), they did not always evoke the same effects on conventional measures of task performance (e.g., IL inactivation reduced the number of reversals while mOFC inactivation reduced the fraction of rewarded trials). In an attempt to understand these apparent discrepancies, we simulated data of 650,250 probabilistic reversal learning sessions (13,005 conditions × 50 simulations) with the earlier used Q-learning model. For visualization purposes, and because PFC inactivations did not affect explore/exploit behavior, *β* was fixed at a value of 1.686 (the average of all animals across all conditions in Fig. 3), but values of learning rates *α*^+^ and *α*^−^ and stickiness parameter *π* were varied; heatmaps of simulated data are presented in Fig. 5.

These simulations demonstrate complex, interacting relationships between the computational model’s parameter values and the four conventional measures of task performance. For example, for this level of *β* (1.686), the number of reversals is highly dependent on the value of the stickiness parameter, but only when both reward and punishment learning rates are high. In addition, the number of rewarded trials requires high learning and is the highest for stickiness parameter values close to 0. Furthermore, win-stay and lose-switch measures are most strongly dependent on stickiness, and also drop dramatically when learning rates become very low (< 0.3). Importantly, the link between win-stay behavior and reward learning versus lose-switch and punishment learning, often assumed in scientific literature (Bari et al. 2010), does not seem as straightforward, as reward and punishment learning rates both affect win-stay and lose-switch behavior. Importantly, it can be seen that a wide range of parameter values allows the animal to perform well in the task, and that, dependent on baseline values of the parameters, decreases of learning rate up to 50% are required to see changes in the conventional measures of task performance. Finally, it should be noted that these heatmaps look different for different values of the explore/exploit parameter *β* (Online Resource 6). For example, during high exploitation of value (high *β*), a higher number of reversals will be obtained with lower values of punishment learning—this is likely due to the notion that in this case it is profitable to ignore false-negative data (i.e., the occasional reward omission) at the high-probability nose poke hole.

## Discussion

In this study, we assessed the effects of pharmacological inactivation of five different subregions of the rat PFC on performance in a probabilistic reversal learning task and used computational modeling to assess how these inactivations disrupted the structure of responding in the task. We found that inactivation of the PrL, IL, mOFC, and lOFC impaired task performance and that this was driven by reductions in punishment learning and response persistence (IL and mOFC) or in a combined reduction in reward and punishment learning (PrL and lOFC). Inactivation of the ACC did not affect conventional performance measures or any of the computational model parameters. It is important to note that only for the stickiness parameter, the two-way ANOVA yielded a significant brain region × interaction effect, suggesting a differential involvement of PFC subregions in response persistence. In contrast, the ANOVA on positive (reward) and negative (reward omission) feedback learning only yielded a main effect of inactivation, suggesting a general reduction in learning after PFC inactivation, regardless of brain region.

Our experimental design was comparable to the study of Dalton et al. (2016), and our findings were, to a large extent, consistent. That is, we found no effects of ACC inactivation on probabilistic reversal learning performance, and we observed changes in performance after inactivation of the PrL, mOFC, and lOFC. There are, however, two important differences: (1) we found a reduction in performance after IL inactivation, whereas Dalton et al. found no effects, and (2) Dalton et al. observed an improvement in reversal learning performance after PrL inactivation, while we observed an impairment. We think that the most likely explanation for these discrepancies arises from baseline differences in behavior. In our simulated data (Fig. 5 and Online Resource 6), we show that a reduction in punishment learning and stickiness (as we observed after IL inactivation) does not necessarily change the number of reversals—it only does so if the effects are strong enough and when baseline functioning allows it. Indeed, there is a wide range of parameter values that is optimal for task performance (both in terms of number of reversals and rewarded trials; see the large spread of the 90th percentile border in Fig. 5). Likewise, reductions in the computational model parameter values may in some cases even lead to an increase in performance, as was observed by Dalton et al. (2016) (see heatmaps for high *β* values in Online Resource 6). Thus, whether changes in the model parameter values lead to changes in conventional behavioral measures of task performance is not straightforward and depends on the characteristics of the animals and the amount of training (i.e., baseline levels of the computational models parameter values). An alternative explanation for the discrepancies between the two studies is that subtle, but important methodological differences exist. For example, in our study, the task was self-paced, so that task performance had more direct consequences for the amount of reward received. Moreover, trial outcomes (either wins or losses) were more explicitly signaled by pertinent cues in our task version. As a result, in our study, task performance may have relied to a greater extent on PrL and IL function, because of their complementary roles in action-outcome tracking (Corbit and Balleine 2003; Killcross and Coutureau 2003) and cue-driven reward pursuit and consumption (Ishikawa et al. 2008; Burgos-Robles et al. 2013).

The simulated data in Fig. 5 and Online Resource 6 give two additional important insights into the data. First, it may explain why similar effects on the computational model parameters sometimes have differential effects on the conventional measures of task performance. For example, both IL and mOFC inactivation reduced punishment learning and stickiness (Fig. 3), whereas IL inactivation reduced the number of reversals and mOFC inactivation decreased the fraction of rewarded trials (Fig. 1c). One observation from the simulated data (Fig. 5 and Online Resource 6) is that all of the computational model parameters influence all of the behavioral measures of task performance, and it depends on the baseline values of these parameters and the size of the effects as of which conventional task parameter is affected. Second, win-stay and lose-switch measures, classically used as a proxy for reward and punishment learning, respectively (Bari et al. 2010), are not specific to either types of learning, as win-stay and lose-switch are both dependent on reward and punishment learning. In fact, win-stay and lose-switch appear more strongly influenced by stickiness parameter *π*, rather than by learning. This finding indicates that win-stay and lose-switch behavior should not be used as straightforward descriptors of sensitivity to reward and punishment, as we have shown before (Verharen et al. 2018).

Together, our data suggest that value-based behaviors in the rat are governed through distinct, but functionally overlapping PFC regions by mediating different aspects of value-based learning and decision-making (Fig. 4). Given this overlap in function, we speculate that within the rat PFC, there is redundant coding of value-related signals. This redundancy could be indicative of the existence of a neural safety net that ensures that essential cognitive operations can continue if activity in a part of the PFC is impaired, for example by neurological disease, pharmacological insults, or stress. Alternatively, there may be coding of value-based learning functions across a larger, interconnected network that eventually mediates decision-making. This suggests distributed, parallel processing of value-related information across different brain circuits, as has been proposed by recent theories (Cisek 2012; Rushworth et al. 2012; Hunt and Hayden 2017). Dissecting these circuits, including the identification of the cell types and neuronal projections that mediate value-based processes across the PFC, is an important topic for future research. Of special interest would be the elucidation of neural pathways that are specialized in subcomponents of learning and decision-making, using contemporary viral vector–based techniques, as has been done recently for projections from the OFC (Groman et al. 2019).

Deficits in reversal learning after pharmacological inactivation or lesion of regions of the PFC have been observed before in different species, although the effects of neural manipulations of the medial PFC (PrL/IL) have been inconsistent (Izquierdo et al. 2017). Overall, it has been suggested that the medial PFC becomes engaged in reversal learning only when the task becomes more complex and requires higher levels of attention (Izquierdo et al. 2017), for example when reward contingencies are probabilistic. Indeed, most studies that have assessed the role of the medial PFC in reversal learning—with for the most part negative results—have used a deterministic version of the task, in which reward contingencies are absolute (i.e., one response always, the other never rewarded) (see Izquierdo et al. 2017). Animals can then rely on more heuristic strategies to perform the task, such as win-stay/lose-switch (Posch 1999), rather than by actively tracking the outcome of the choice options over time, which may require a lesser involvement of PFC-mediated value-based processes. Indeed, other behavioral tasks that have been shown to rely on functional activity of the PFC, such as set shifting (Birrell and Brown 2000) and probabilistic discounting (St Onge and Floresco 2010), are more complex by nature and involve changes in behavioral strategies, reward contingency switches, and/or probabilistic reward delivery. It may therefore be the case that our results extend beyond probabilistic reversal learning and that the behavioral effects of PFC lesion or inactivation in these tasks are the result of general changes in processes underlying value updating and decision-making, including reward learning, punishment learning, and/or choice perseveration.

The OFC is thought to be important for a variety of value-based decision-making processes, with functional heterogeneity along both the mediolateral and the anteroposterior axes (Izquierdo 2017). In the present study, we mainly targeted the anterior MO/VO region of the mOFC and the more dorsal part of the VO/LO region of the lOFC, which have been implicated in functions such as decision-making under uncertainty and outcome prediction (Izquierdo 2017). Although impaired decision-making under uncertainty does capture the deficits in conventional measures of reversal learning that we observed after lOFC inactivation, its role in reward and punishment learning suggests a broader functionality, for example covered by the theory that the lOFC keeps a cognitive map of task structure (Rudebeck and Murray 2014; Wilson et al. 2014). The inability of animals to generate such a cognitive map after inactivation may lead to general disruptions in behavior that in the behavioral model are best described by the inability to adapt to reward and punishment. Thus, “model-free” reinforcement learning models, as the one used in this study, may not capture the true function of the lOFC, and the observed learning deficit after its inactivation may in reality be due to the disruptions in higher-order cognitive processes. In line with this notion is the finding that mOFC inactivation evoked qualitative changes in task strategy, as the model fitting procedure on the mOFC inactivation sessions showed that the Rescorla-Wagner-Pearce-Hall best described those sessions, rather than the Rescorla-Wagner model that best described the baseline sessions (Online Resource 5). That said, assessing how inactivation affects the structure of responding in the task may provide important clues about how this region contributes to complex decision-making behavior, being it directly involved in the component processes of model-free decision-making behavior or not.

A recent study showed changes in positive and negative feedback learning, as well as in response persistence (stickiness), in people with stimulant abuse disorder and obsessive-compulsive disorder (Kanen et al. 2019), two psychopathologies in which the human PFC has been implicated (Bechara and Van Der Linden 2005; Volkow and Morales 2015). This may provide interesting clinical relevance to our findings, especially since these same authors provided a potential drug target (the dopamine D_2/3_ receptor) for modulating these components of learning and decision-making in humans (Kanen et al. 2019). In addition, the subregion-specific involvement of the mOFC and PrL in stickiness may be of special clinical importance, given the suggested involvement of maladaptive response persistence—irrespective of outcome—in addictive behaviors (Everitt and Robbins 2016).

### Concluding remarks

Overall, our study reveals a rat PFC that is anatomically organized into functional districts, in which each function supporting probabilistic reversal learning depends on activity in at least two different PFC subregions. Such a topographic map of PFC function suggests an intricate balance between an efficient distribution of function, so that not all regions are engaged in all aspects of task behavior, and safeguarding of function, so that each function relies on activity in at least two brain regions. Interestingly, punishment learning was dependent on four of five PFC regions, suggesting that negative feedback learning is especially robustly integrated in the frontal lobe, perhaps because of its importance for survival. Altogether, we demonstrate a specialized but overlapping functional-anatomical organization of higher-order cognition within the rat PFC, providing important insights into the functional architecture of the mammalian brain.

## Electronic supplementary material

ESM 1 (PDF 1130 kb)

## Data Availability

The entire dataset, including scripts of the simulated data, is openly available at github.com/jeroenphv.

## References

[CR1] Bari A (2010). Serotonin modulates sensitivity to reward and negative feedback in a probabilistic reversal learning task in rats. Neuropsychopharmacology.

[CR2] Bechara A, Van Der Linden M (2005). Decision-making and impulse control after frontal lobe injuries. Curr Op Neurol.

[CR3] Birrell JM, Brown VJ (2000). Medial frontal cortex mediates perceptual attentional set shifting in the rat. J Neurosci.

[CR4] Burgos-Robles A, Bravo-Rivera H, Quirk GJ (2013). Prelimbic and infralimbic neurons signal distinct aspects of appetitive instrumental behavior. PLoS One.

[CR5] Chudasama Y, Robbins TW (2003). Dissociable contributions of the orbitofrontal and infralimbic cortex to Pavlovian autoshaping and discrimination reversal learning: further evidence for the functional heterogeneity of the rodent frontal cortex. J Neurosci.

[CR6] Cisek P (2012). Making decisions through a distributed consensus. Curr Opin Neurobiol.

[CR7] Cohen JD, McClure SM, Yu AJ (2007). Should I stay or should I go? How the human brain manages the trade-off between exploitation and exploration. Philos Trans R Soc Lond Ser B Biol Sci.

[CR8] Corbit LH, Balleine BW (2003). The role of prelimbic cortex in instrumental conditioning. Behav Brain Res.

[CR9] Dalley JW, Cardinal RN, Robbins TW (2004). Prefrontal executive and cognitive functions in rodents: neural and neurochemical substrates. Neurosci Biobehav Rev.

[CR10] Dalton GL, Wang NY, Phillips AG, Floresco SB (2016). Multifaceted contributions by different regions of the orbitofrontal and medial prefrontal cortex to probabilistic reversal learning. J Neurosci.

[CR11] Dayan P, Daw ND (2008). Decision theory, reinforcement learning, and the brain. Cogn Affect Behav Neurosci.

[CR12] Everitt BJ, Robbins TW (2016). Drug addiction: updating actions to habits to compulsions ten years on. Annu Rev Psychol.

[CR13] Floresco SB (2013). Prefrontal dopamine and behavioral flexibility: shifting from an “inverted-U” toward a family of functions. Front Neurosci.

[CR14] Gershman SJ (2016). Empirical priors for reinforcement learning models. J Math Psychol.

[CR15] Groman SM, Keistler C, Keip AJ, Hammarlund E, DiLeone RJ, Pittenger C, Lee D, Taylor JR (2019). Orbitofrontal circuits control multiple reinforcement-learning processes. Neuron.

[CR16] Hervig ME, Fiddian L, Piilgaard L, Božič T, Blanco-Pozo M, Knudsen C, Olesen SF, Alsiö J, Robbins TW (2019) Dissociable and paradoxical roles of rat medial and lateral orbitofrontal cortex in visual serial reversal learning. Cereb Cortex. 10.1093/cercor/bhz144 10.1093/cercor/bhz144 PMC7132932 31343680

[CR17] Hornak J, O'doherty J, Bramham J, Rolls E, Morris R, Bullock P, Polkey C (2004). Reward-related reversal learning after surgical excisions in orbito-frontal or dorsolateral prefrontal cortex in humans. J Cogn Neurosci.

[CR18] Hunt LT, Hayden BY (2017). A distributed, hierarchical and recurrent framework for reward-based choice. Nat Rev Neurosci.

[CR19] Ishikawa A, Ambroggi F, Nicola SM, Fields HL (2008). Contributions of the amygdala and medial prefrontal cortex to incentive cue responding. Neuroscience.

[CR20] Izquierdo A (2017). Functional heterogeneity within rat orbitofrontal cortex in reward learning and decision making. J Neurosci.

[CR21] Izquierdo A, Brigman JL, Radke AK, Rudebeck PH, Holmes A (2017). The neural basis of reversal learning: an updated perspective. Neuroscience.

[CR22] Kanen JW, Ersche KD, Fineberg NA, Robbins TW, Cardinal RN (2019). Computational modelling reveals contrasting effects on reinforcement learning and cognitive flexibility in stimulant use disorder and obsessive-compulsive disorder: remediating effects of dopaminergic D2/3 receptor agents. Psychopharmacology.

[CR23] Killcross S, Coutureau E (2003). Coordination of actions and habits in the medial prefrontal cortex of rats. Cereb Cortex.

[CR24] Li J, Schiller D, Schoenbaum G, Phelps EA, Daw ND (2011). Differential roles of human striatum and amygdala in associative learning. Nat Neurosci.

[CR25] Miller EK, Cohen JD (2001). An integrative theory of prefrontal cortex function. Annu Rev Neurosci.

[CR26] Pearce JM, Hall G (1980). A model for Pavlovian learning: variations in the effectiveness of conditioned but not of unconditioned stimuli. Psychol Rev.

[CR27] Posch M (1999). Win–stay, lose–shift strategies for repeated games—memory length, Aspiration Levels and Noise. J Theor Biol.

[CR28] Rescorla RA, Wagner AR (1972). A theory of Pavlovian conditioning: variations in the effectiveness of reinforcement and nonreinforcement. Classic Condition II: Curr Res Theory.

[CR29] Rigoux L, Stephan KE, Friston KJ, Daunizeau J (2014). Bayesian model selection for group studies - revisited. Neuroimage.

[CR30] Robbins TW, Arnsten AF (2009). The neuropsychopharmacology of fronto-executive function: monoaminergic modulation. Annu Rev Neurosci.

[CR31] Roberts AC (2006). Primate orbitofrontal cortex and adaptive behaviour. Trends Cogn Sci.

[CR32] Rudebeck PH, Murray EA (2014). The orbitofrontal oracle: cortical mechanisms for the prediction and evaluation of specific behavioral outcomes. Neuron.

[CR33] Rushworth MF, Kolling N, Sallet J, Mars RB (2012). Valuation and decision-making in frontal cortex: one or many serial or parallel systems?. Curr Opin Neurobiol.

[CR34] Schultz W, Dayan P, Montague PR (1997). A neural substrate of prediction and reward. Science.

[CR35] St Onge JR, Floresco SB (2010). Prefrontal cortical contribution to risk-based decision making. Cereb Cortex.

[CR36] Sutton RS, Barto AG (1998) Reinforcement learning: an introduction. MIT press, Cambridge, MA

[CR37] Verharen JPH (2018). A neuronal mechanism underlying decision-making deficits during hyperdopaminergic states. Nat Commun.

[CR38] Verharen JPH, Adan RAH, Vanderschuren LJMJ (2019). Differential contributions of striatal dopamine D1 and D2 receptors to component processes of value-based decision making. Neuropsychopharmacology.

[CR39] Verharen JPH, Adan RAH, Vanderschuren LJMJ (2019b) How Reward and aversion shape motivation and decision making: a computational account. Neuroscientist. 26:87–99 10.1177/1073858419834517 30866712

[CR40] Verharen JPH, Kentrop J, Vanderschuren LJMJ, Adan RAH (2019c) Reinforcement learning across the rat estrous cycle. Psychoneuroendocrinology 100:27–31 10.1016/j.psyneuen.2018.09.016 30273796

[CR41] Volkow ND, Morales M (2015). The brain on drugs: from reward to addiction. Cell.

[CR42] Wilson RC, Takahashi YK, Schoenbaum G, Niv Y (2014). Orbitofrontal cortex as a cognitive map of task space. Neuron.

