# Supplementary material for: Modulation of value-based decision making behavior by subregions of the rat prefrontal cortex
Source: Psychopharmacology (Berl). 2020 Feb 6;237(5):1267–80. doi: 10.1007/s00213-020-05454-7 (PMC7196947; doi:10.1007/s00213-020-05454-7)

Electronic Supplementary Material

Modulation of value-based decision making behavior by subregions of the rat prefrontal cortex

Jeroen P.H. Verharen[1,2,3], Hanneke E.M. den Ouden[4], Roger A.H. Adan[1,5], Louk J.M.J. Vanderschuren[2,5,*]

[1] Brain Center Rudolf Magnus, Department of Translational Neuroscience, University Medical Center Utrecht, 3584CG Utrecht, The Netherlands.

[2] Department of Animals in Science and Society, Division of Behavioural Neuroscience, Faculty of Veterinary Medicine, Utrecht University, 3584CM Utrecht, The Netherlands.

[3] Helen Wills Neuroscience Institute, Department of Molecular and Cell Biology, University of California Berkeley, Berkeley, CA 94720, United States

[4] Donders Institute for Brain, Cognition and Behaviour, Radboud University, 6525HR Nijmegen, The Netherlands.

[5] These authors contributed equally.

* Corresponding author: l.j.m.j.vanderschuren@uu.nl, +31-30-2535239

Online Resource 1

Supplementary Statistics Table

Figure	Test used	*n*	p value	Test statistic	Effect size<br>(Partial eta squared)
1c<br>Reversals	2-way repeated measures ANOVA	49 rats	Main drug effect<br>p = 0.0006***<br><br>Main group effect<br>p = 0.0003***<br><br>Group * drug interaction<br>p = 0.1252	Main drug effect<br>F(1, 44) = 13.63<br><br>Main group effect<br>F(4, 44) = 6.469<br><br>Group * drug interaction<br>F(4, 44) = 1.913	
	post-hoc Holm-Sidak ACC group (sal vs BM)	10 rats	p = 0.9931	t(44) = 0.009	Measurement 1 = 0.1615<br>Measurement 2 = 0.1380<br>Average = 0.0000
	post-hoc Holm-Sidak<br>PrL group (sal vs BM)	12 rats	p = 0.1636	t(44) = 1.948	Measurement 1 = 0.0846<br>Measurement 2 = 0.4889<br>Average = 0.3676
	post-hoc Holm-Sidak<br>IL group (sal vs BM)	9 rats	p = 0.0285 *	t(44) = 2.818	Measurement 1 = 0.3291<br>Measurement 2 = 0.1913<br>Average = 0.5022
	post-hoc Holm-Sidak<br>mOFC group (sal vs BM)	9 rats	p = 0.8780	t(44) = 0.4559	Measurement 1 = 0.4254<br>Measurement 2 = 0.0705<br>Average = 0.0361
	post-hoc Holm-Sidak<br>lOFC group (sal vs BM)	9 rats	p = 0.0215 *	t(44) = 3.007	Measurement 1 = 0.0954<br>Measurement 2 = 0.5068<br>Average = 0.3506
1c<br>Rewarded	2-way repeated measures ANOVA	49 rats	Main drug effect<br>p < 0.0001 ****<br><br>Main group effect<br>p = 0.0174 *<br><br>Group * drug interaction<br>p = 0.4666	Main drug effect<br>F(1, 44) = 21.80<br><br>Main group effect<br>F(4, 44) = 3.362<br><br>Group * drug interaction<br>F(4, 44) = 0.9099	
	post-hoc Holm-Sidak ACC group (sal vs BM)	10 rats	p = 0.3814	t(44) = 1.110	Measurement 1 = 0.6551<br>Measurement 2 = 0.0699<br>Average = 0.1992
	post-hoc Holm-Sidak<br>PrL group (sal vs BM)	12 rats	p = 0.0414 *	t(44) = 2.672	Measurement 1 = 0.1703<br>Measurement 2 = 0.3810<br>Average = 0.4303
	post-hoc Holm-Sidak<br>IL group (sal vs BM)	9 rats	p = 0.3814	t(44) = 1.262	Measurement 1 = 0.0023<br>Measurement 2 = 0.0898<br>Average = 0.1157

Figure	Test used	*n*	p value	Test statistic	Effect size<br>(Partial eta squared)
	post-hoc Holm-Sidak<br>mOFC group (sal vs BM)	9 rats	$p = 0.0071$**	$t(44) = 3.402$	Measurement 1 = 0.1153<br>Measurement 2 = 0.5617<br>Average = 0.6077
	post-hoc Holm-Sidak<br>lOFC group (sal vs BM)	9 rats	$p = 0.1367$	$t(44) = 2.036$	Measurement 1 = 0.0573<br>Measurement 2 = 0.2831<br>Average = 0.2941
1c<br>Trials complete d	2-way repeated measures ANOVA	49 rats	Main drug effect<br>$p = 0.3012$<br><br>Main group effect<br>$p = 0.0026$<br><br>Group * drug interaction<br>$p = 0.3942$	Main drug effect<br>$F(1, 45) = 1.094$<br><br>Main group effect<br>$F(4, 45) = 4.791$<br><br>Group * drug interaction<br>$F(4, 45) = 1.046$	
1c<br>Response latency	2-way repeated measures ANOVA	49 rats	Main drug effect<br>$p = 0.7208$<br><br>Main group effect<br>$p = 0.0709$<br><br>Group * drug interaction<br>$p = 0.9479$	Main drug effect<br>$F(1, 44) = 0.1294$<br><br>Main group effect<br>$F(4, 44) = 2.328$<br><br>Group * drug interaction<br>$F(4, 44) = 0.1793$	
3<br>Reward learning	2-way repeated measures ANOVA	49 rats	Main drug effect<br>$p < 0.0001$****<br><br>Main group effect<br>$p = 0.0002$***<br><br>Group * drug interaction<br>$p = 0.5832$	Main drug effect<br>$F(1, 44) = 20.40$<br><br>Main group effect<br>$F(4, 44) = 7.020$<br><br>Group * drug interaction<br>$F(4, 44) = 0.7195$	
	post-hoc Holm-Sidak ACC group (sal vs BM)	10 rats	$p = 0.3578$	$t(44) = 1.305$	Measurement 1 = 0.1450<br>Measurement 2 = 0.1148<br>Average = 0.1672
	post-hoc Holm-Sidak<br>PrL group (sal vs BM)	12 rats	$p = 0.0111$*	$t(44) = 3.246$	Measurement 1 = 0.1065<br>Measurement 2 = 0.6025<br>Average = 0.5714
	post-hoc Holm-Sidak<br>IL group (sal vs BM)	9 rats	$p = 0.3578$	$t(44) = 1.036$	Measurement 1 = 0.0000<br>Measurement 2 = 0.2580<br>Average = 0.0923
	post-hoc Holm-Sidak<br>mOFC group (sal vs BM)	9 rats	$p = 0.1466$	$t(44) = 2.002$	Measurement 1 = 0.1956<br>Measurement 2 = 0.1571<br>Average = 0.2475
	post-hoc Holm-Sidak<br>lOFC group (sal vs BM)	9 rats	$p = 0.0435$*	$t(44) = 2.653$	Measurement 1 = 0.3670<br>Measurement 2 = 0.4936<br>Average = 0.5913

Figure	Test used	*n*	p value	Test statistic	Effect size (Partial eta squared)
3 Punish-ment learning	2-way repeated measures ANOVA	49 rats	Main drug effect $p < 0.0001$**** <br>Main group effect $p < 0.0001$**** <br>Group * drug interaction $p = 0.1345$	Main drug effect $F(1, 44) = 44.63$ <br>Main group effect $F(4, 44) = 8.281$ <br>Group * drug interaction $F(4, 44) = 1.860$	
	post-hoc Holm-Sidak ACC group (sal vs BM)	10 rats	$p = 0.3798$	$t(44) = 0.8871$	Measurement 1 = 0.0251<br>Measurement 2 = 0.0486<br>Average = 0.0614
	post-hoc Holm-Sidak PrL group (sal vs BM)	12 rats	$p = 0.0007$***	$t(44) = 4.179$	Measurement 1 = 0.4016<br>Measurement 2 = 0.5421<br>Average = 0.6335
	post-hoc Holm-Sidak IL group (sal vs BM)	9 rats	$p = 0.0011$**	$t(44) = 3.945$	Measurement 1 = 0.2946<br>Measurement 2 = 0.4343<br>Average = 0.5327
	post-hoc Holm-Sidak mOFC group (sal vs BM)	9 rats	$p = 0.0497$*	$t(44) = 2.318$	Measurement 1 = 0.6776<br>Measurement 2 = 0.2121<br>Average = 0.6304
	post-hoc Holm-Sidak lOFC group (sal vs BM)	9 rats	$p = 0.0019$**	$t(44) = 3.382$	Measurement 1 = 0.4988<br>Measurement 2 = 0.6789<br>Average = 0.7280
3 Stickiness	2-way repeated measures ANOVA	49 rats	Main drug effect $p = 0.0005$*** <br>Main group effect $p = 0.2508$ <br>Group * drug interaction $p = 0.0137$*	Main drug effect $F(1, 44) = 14.07$ <br>Main group effect $F(4, 44) = 1.397$ <br>Group * drug interaction $F(4, 44) = 3.540$	
	post-hoc Holm-Sidak ACC group (sal vs BM)	10 rats	$p = 0.9494$	$t(44) = 0.3050$	Measurement 1 = 0.0440<br>Measurement 2 = 0.0976<br>Average = 0.0261
	post-hoc Holm-Sidak PrL group (sal vs BM)	12 rats	$p = 0.9494$	$t(44) = 0.4848$	Measurement 1 = 0.0001<br>Measurement 2 = 0.1298<br>Average = 0.0174
	post-hoc Holm-Sidak IL group (sal vs BM)	9 rats	$p = 0.0010$**	$t(44) = 4.046$	Measurement 1 = 0.3641<br>Measurement 2 = 0.2258<br>Average = 0.6792
	post-hoc Holm-Sidak mOFC group (sal vs BM)	9 rats	$p = 0.0115$*	$t(44) = 3.157$	Measurement 1 = 0.6981<br>Measurement 2 = 0.0675<br>Average = 0.6443
	post-hoc Holm-Sidak lOFC group (sal vs BM)	9 rats	$p = 0.9494$	$t(44) = 0.1775$	Measurement 1 = 0.0040<br>Measurement 2 = 0.0113<br>Average = 0.0022

Figure	Test used	*n*	p value	Test statistic	Effect size<br>(Partial eta squared)
3<br>Explore/<br>exploit	2-way repeated measures ANOVA	49 rats	Main drug effect<br>p = 0.8299<br><br>Main group effect<br>p = 0.1193<br><br>Group * drug interaction<br>p = 0.2838	Main drug effect<br>F(1, 44) = 0.04671<br><br>Main group effect<br>F(4, 44) = 1.948<br><br>Group * drug interaction<br>F(4, 44) = 1.303	

Online Resource 2

Table containing the equations of the different models

Model	Free parameters	Learning model	Observation equation
RW1	α, β	$Q_{s,t} = \begin{cases} Q_{s,t-1} + \alpha \cdot \text{RPE}_t & \text{for win trials} \\ Q_{s,t-1} + \alpha \cdot \text{RPE}_t & \text{for lose trials} \end{cases}$	$p_{right,t} = \frac{\exp(\beta \cdot Q_{right,t})}{\exp(\beta \cdot Q_{left,t}) + \exp(\beta \cdot Q_{right,t})}$
RW2	α+, α-, β	$Q_{s,t} = \begin{cases} Q_{s,t-1} + \alpha^{+} \cdot \text{RPE}_t & \text{for win trials} \\ Q_{s,t-1} + \alpha^{-} \cdot \text{RPE}_t & \text{for lose trials} \end{cases}$	$p_{right,t} = \frac{\exp(\beta \cdot Q_{right,t})}{\exp(\beta \cdot Q_{left,t}) + \exp(\beta \cdot Q_{right,t})}$
RW3	α+, α-, β, π	$Q_{s,t} = \begin{cases} Q_{s,t-1} + \alpha^{+} \cdot \text{RPE}_t & \text{for win trials} \\ Q_{s,t-1} + \alpha^{-} \cdot \text{RPE}_t & \text{for lose trials} \end{cases}$	$p_{right,t} = \frac{\exp(\beta \cdot Q_{right,t} + \pi \cdot \phi_{right,t})}{\exp(\beta \cdot Q_{left,t} + \pi \cdot \phi_{left,t}) + \exp(\beta \cdot Q_{right,t} + \pi \cdot \phi_{right,t})}$
RW-PH	α, β, π, η	$Q_{s,t} = \begin{cases} Q_{s,t-1} + \alpha \cdot \gamma_t \cdot \text{RPE}_t & \text{for win trials} \\ Q_{s,t-1} + \alpha \cdot \gamma_t \cdot \text{RPE}_t & \text{for lose trials} \end{cases}$ with $\gamma_t = \eta \cdot \lvert \text{RPE}_t \rvert + (1-\eta) \cdot \gamma_{t-1}$	$p_{right,t} = \frac{\exp(\beta \cdot Q_{right,t} + \pi \cdot \phi_{right,t})}{\exp(\beta \cdot Q_{left,t} + \pi \cdot \phi_{left,t}) + \exp(\beta \cdot Q_{right,t} + \pi \cdot \phi_{right,t})}$

In this table, α = Rescorla-Wagner learning rate, β = choice stochasticity, π = stickiness factor, η = Pearce-Hall associability factor, $Q_{s,t}$ = value of nose poke *s* on trial *t*, $p_{s,t}$ = choice probability of nose poke *s* on trial *t*, ϕ = boolean that is 1 if nose poke *s* is chosen on the previous trial and 0 if unchosen on previous trial, RPE = reward prediction error, and γ_t = associability on trial *t*.

Online Resource 3

Infusion locations

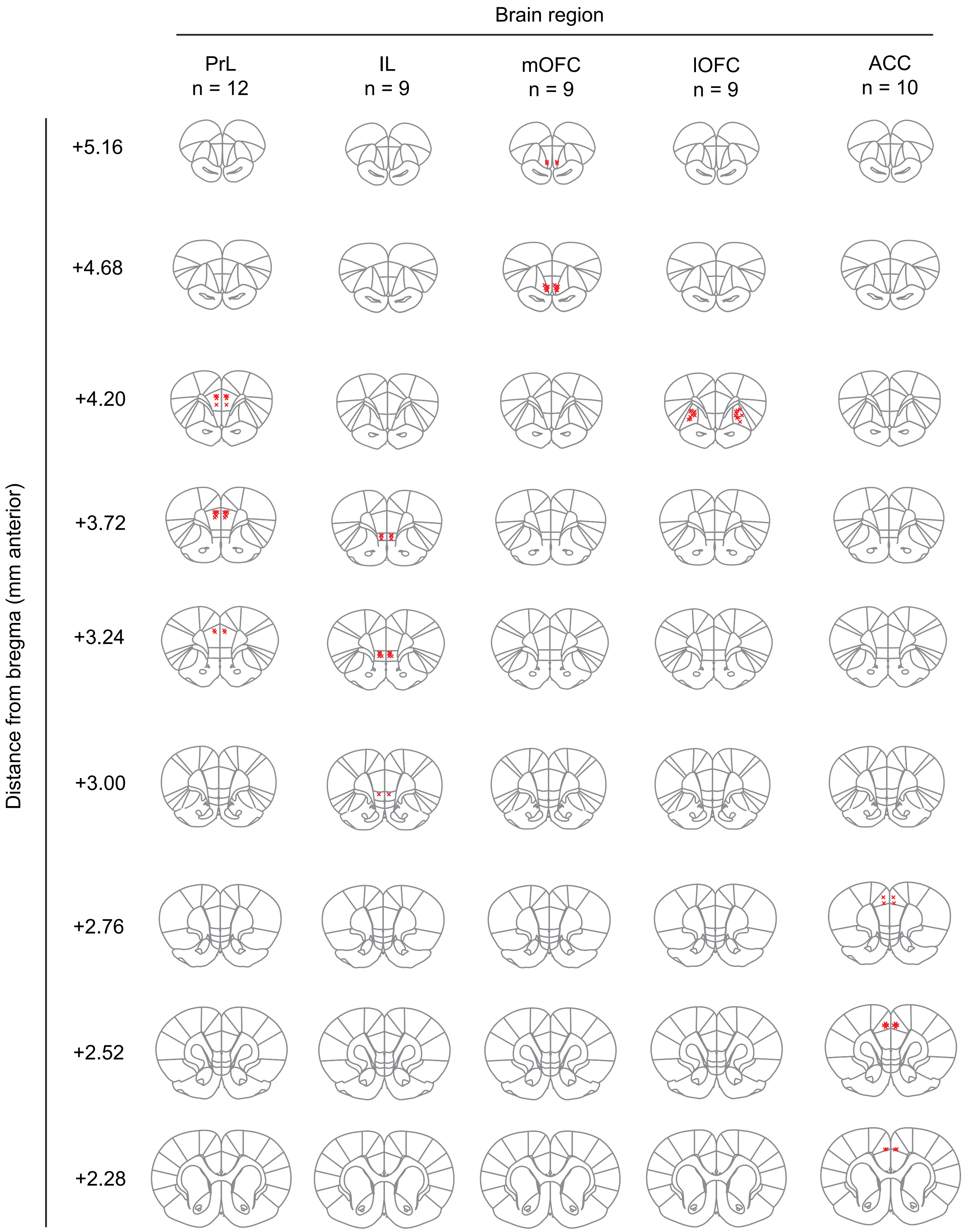

Online Resource 4

Model selection

	Model	Free parameters	Aggregate LL	$P_{explained}$	Aggregate AIC	Aggregate BIC	# of sessions best described by model	XP	PXP
1	Random	-	-32757	0.5000	65514	65514	0/196	0	0
2	Rescorla-Wagner 1	α, β	-28815	0.5435	58414	59761	40/196	0	0
3	Rescorla-Wagner 2	α+, α-, β	-28415	0.5481	58007	60026	25/196	0	0
4	Rescorla-Wagner 3	α+, α-, β, π	-27818	0.5551	57204	59897	74/196	0.9999	0.9999
5	Rescorla-Wagner-Pearce-Hall hybrid	α, β, π, η	-27973	0.5533	57513	60206	57/196	0.0001	0.0001

Abbreviations: LL, log-likelihood; $P_{explained}$, fraction of choices explained by the model on every single trial (total trials on average ≈ 241); AIC, Akaike Information Criterion; BIC, Bayesian Information Criterion; XP, exceedance probability; PXP, protected exceedance probability.

Online Resource 5

Model selection per inactivation condition

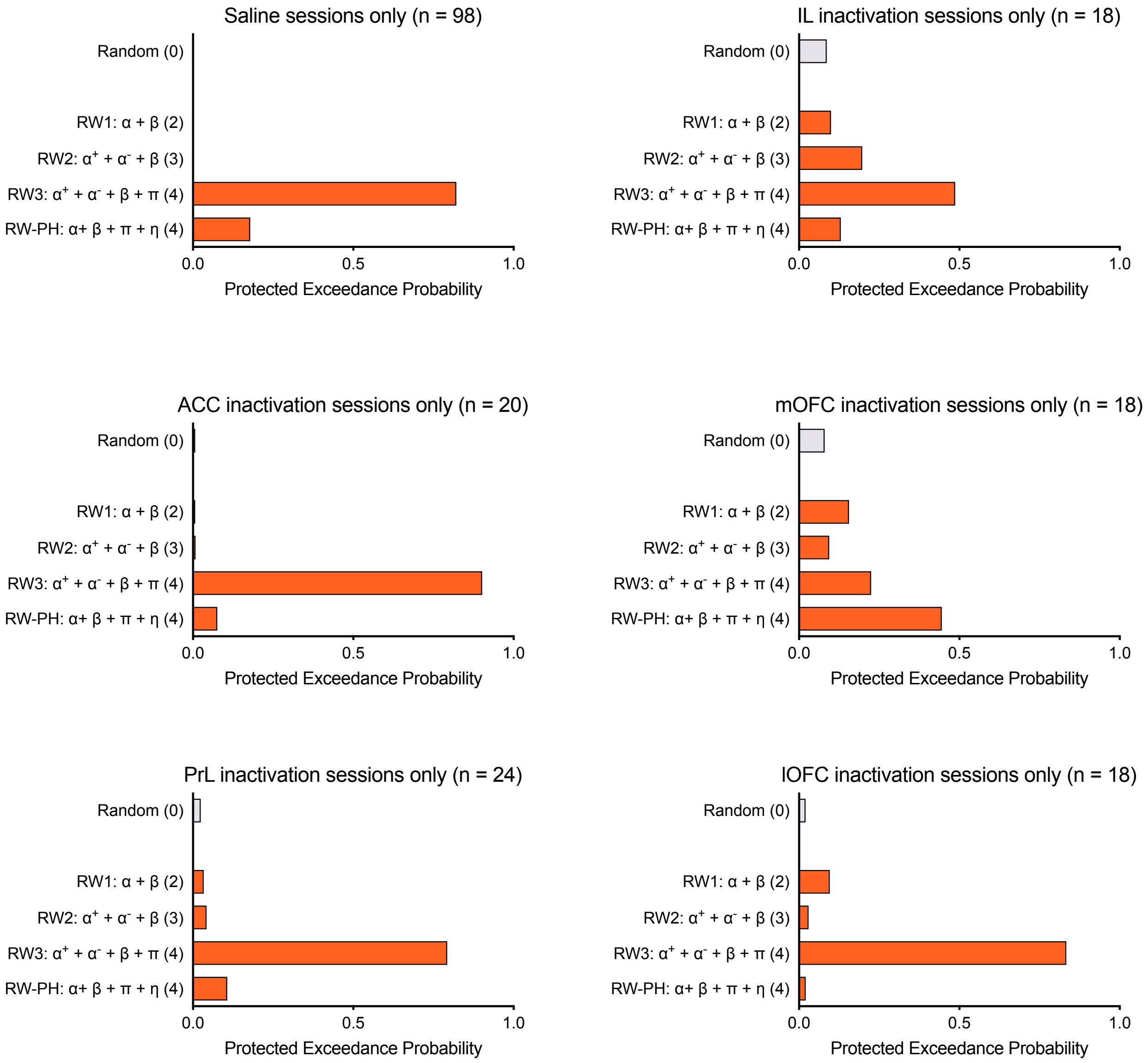

Online Resource 6

Simulated data showing the number of reversals per 100 trials for different values of explore/exploit parameter β

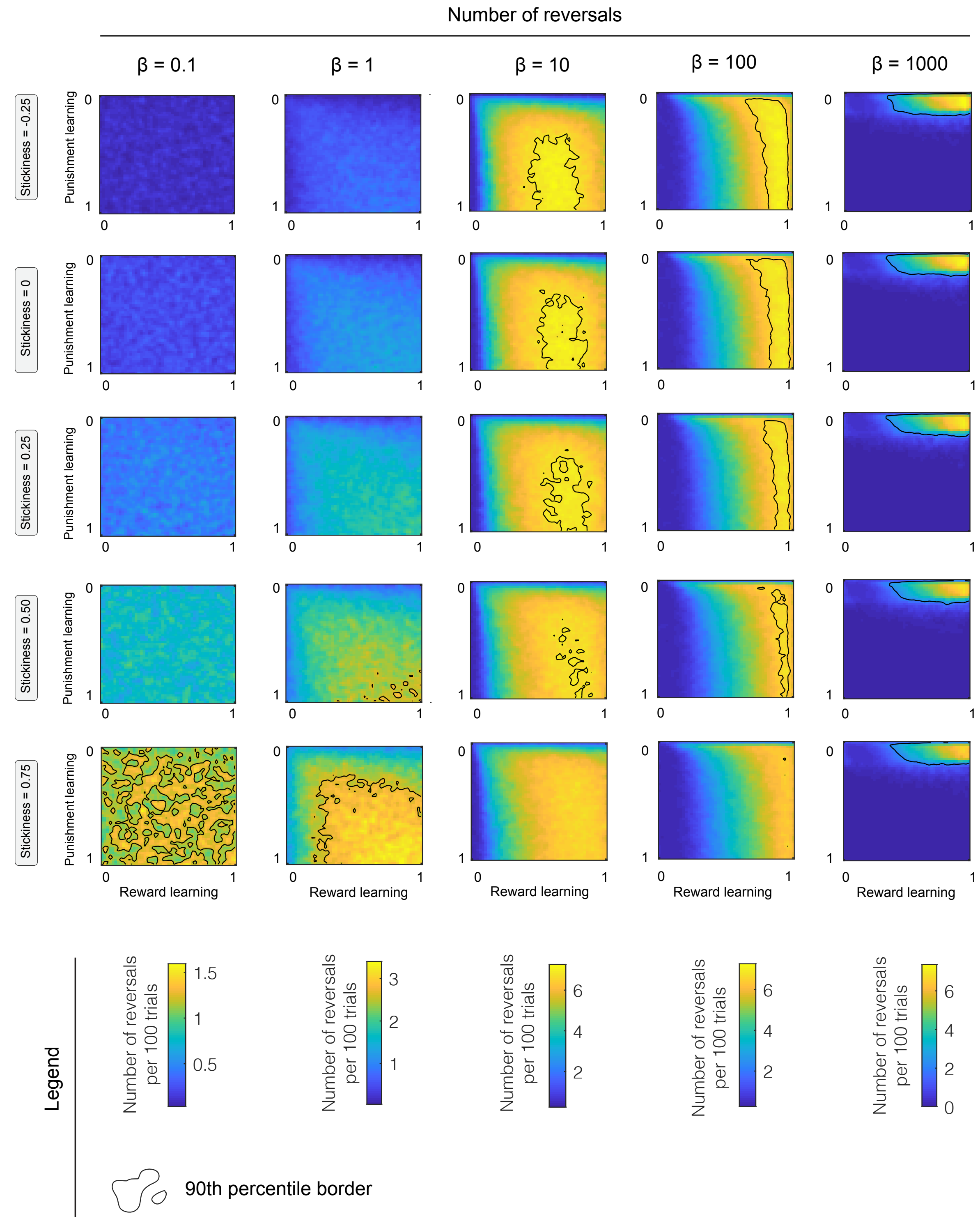

Supplement: Supplementary file 1 — (PDF 1130 kb) [file 213_2020_5454_MOESM1_ESM.pdf]
